# The Mechanisms of Inhibition Effects on Bubble Growth in He-Irradiated 316L Stainless Steel Fabricated by Selective Laser Melting

**DOI:** 10.3390/ma16113922

**Published:** 2023-05-24

**Authors:** Shangkun Shen, Zhangjie Sun, Liyu Hao, Xing Liu, Jian Zhang, Kunjie Yang, Peng Liu, Xiaobin Tang, Engang Fu

**Affiliations:** 1 State Key Laboratory of Nuclear Physics and Technology, Department of Technical Physics, School of Physics, Peking University, Beijing 100871, China; 2 Department of Nuclear Science and Technology, Nanjing University of Aeronautics and Astronautics, Nanjing 211106, Chinatangxiaobin@nuaa.edu.cn (X.T.); 3 College of Energy, Xiamen University, Xiamen 361005, China; 4 College of Nuclear Equipment and Nuclear Engineering, Yantai University, Yantai 264005, China; 5 Key Laboratory of Particle Physics and Particle Irradiation (MOE), Institute of Frontier and Interdisciplinary Science, Shandong University, Qingdao 266237, China; pengliu@sdu.edu.cn

**Keywords:** selective laser melting (SLM), AISI 316L stainless steel, helium bubble, transmission electron microscope (TEM), electron energy loss spectroscopy (EELS)

## Abstract

The AISI 316L austenitic stainless steel fabricated by selective laser melting (SLM) is considered to have great prospects for applications in nuclear systems. This study investigated the He-irradiation response of SLM 316L, and several possible reasons for the improved He-irradiation resistance of SLM 316L were systematically revealed and evaluated by using TEM and related techniques. The results show that the effects of unique sub-grain boundaries have primary contributions to the decreased bubble diameter in SLM 316L compared to that in the conventional 316L counterpart, while the effects of oxide particles on bubble growth are not the dominant factor in this study. Moreover, the He densities inside the bubbles were carefully measured using electron energy loss spectroscopy (EELS). The mechanism of stress-dominated He densities in bubbles was validated, and the corresponding reasons for the decrease in bubble diameter were freshly proposed in SLM 316L. These insights help to shed light on the evolution of He bubbles and contribute to the ongoing development of the steels fabricated by SLM for advanced nuclear applications.

## 1. Introduction

The development of advanced nuclear power reactors has put forward new requirements for the manufacturing efficiency and refinement of the structural materials [1]. Additive manufacturing (AM) has aroused widespread interest in recent decades due to its advantages of unrivaled design freedom for complex parts and short lead times [2,3]. Among various AM methods, selective laser melting (SLM), as one of the most promising techniques for metal fabrications, allows for the low-cost fabrication of workpieces with high structural integrity [3,4,5]. Thus, the SLM technique can be used not only to optimize the manufacturing process of complex structural components in advanced nuclear reactors, but also to significantly reduce the cost and lead time of the manufacturing materials. In addition, the materials fabricated by SLM have extremely high precision and quality [6,7], which can effectively meet the requirements of structural materials for nuclear applications.

Combining the advantages of the SLM process with the conventional structural materials used in nuclear systems, the SLM 316L stainless steel was proposed with great potential in nuclear applications [7,8,9,10,11]. On one hand, the excellent performance of strength and ductility in SLM 316L has been verified, which is attributed to the unique cellular dislocation structures frequently reported in AM Fe-based alloys [12]. On the other hand, the unique cellular structure in SLM 316L is considered to provide effective sink sites for irradiation-induced defects, and therefore, improve the extra irradiation resistance of SLM 316L compared to that of conventional AISI 316L. For example, Sun et al. [8] demonstrated the great He radiation tolerance of SLM 316L, which is due to the interfaces composed of cellular sub-grains and nano-inclusions. Similarly, the inhibition effects of dislocation structures and oxide-matrix interfaces on bubble evolution were confirmed at elevated temperatures of 800 °C [13]. Furthermore, Song et al. [14] recently reported the improved resistance of irradiation-assisted stress corrosion cracking (IASCC) in hot-isotropic pressed AM 316L compared to that in conventionally forged 316L under proton irradiation. Lin et al. [7] found that the austenite phase in SLM 316L is more stable than that of cold-rolled 316L under heavy-ion irradiation, implying that SLM 316L exhibits excellent phase stability. The above reports have verified the improved irradiation resistance of SLM 316L from many aspects, but the internal mechanisms of the improvement still need to be further integrated and explored. In addition, existing conclusions on irradiated SLM 316L have focused on the effects of unique cellular sub-grains and oxide particles on defect evolution despite the possible stress fields of the matrix induced by rapid melting and cooling rates in the manufacturing process. Therefore, the existing results need to be reintegrated or verified, and other possible factors need to be further clarified in the systematic investigation on the mechanisms of improved irradiation resistance in SLM 316L.

In the present study, the SLM 316L sample and conventional cold-rolled 316L sample were irradiated by 400 keV He^+^ ions to a fluence of 1 × 10^17^ ions/cm^2^ at 450 °C to investigate the irradiation resistance of the two types of 316L. Various characterization techniques, including transmission electron microscopy (TEM) and electron energy-loss spectroscopy (EELS), were used to provide the essential evidence. Finally, the several unique factors of sub-grains, oxide particles, and He densities inside the bubbles were systematically evaluated.

## 2. Material and Methods

Commercial AISI 316L powders were milled with a ball-to-power mass ratio of 5:3 for 24 h by ball milling in the argon atmosphere. During the ball milling treatment, a powder with uniform size (average particle size of about 50 μm) and high sphericity was carefully prepared, as shown in Figure S1 in the Supplementary Material. The obtained powder can ensure the fluidity and is suitable for the subsequent laser melting preparation. The SLM 316L sample was fabricated on the SLM (NCL-M2120 with the maximum power of 500 W) equipment. During the building process, the fixed laser parameters (power of 200 W, scanning of 1100 mm/s, line spacing of 0.06 mm, and spot diameter of 70 μm) were applied to obtain the SLM 316L samples with the highest density and optimal quality. The specific preparation process can be found in a previous study [15]. The conventional 316L bulk was annealed at 1080 °C for 12 h and then ~15% deformed through cold rolling treatment (hereinafter denoted as CR 316L). Due to the small degree of deformation, the CR 316L in this study has formed some twins but does not show typical rolling morphology. The chemical compositions of the two types of 316L can be found in Table 1. Furthermore, the samples were cut into thin slices and electrochemically polished to remove surface stress. The microstructures of the pristine samples were characterized by using scanning electron microscope (SEM, ThermoFisher Quattro S operated at 15 kV).

**Figure 1:**
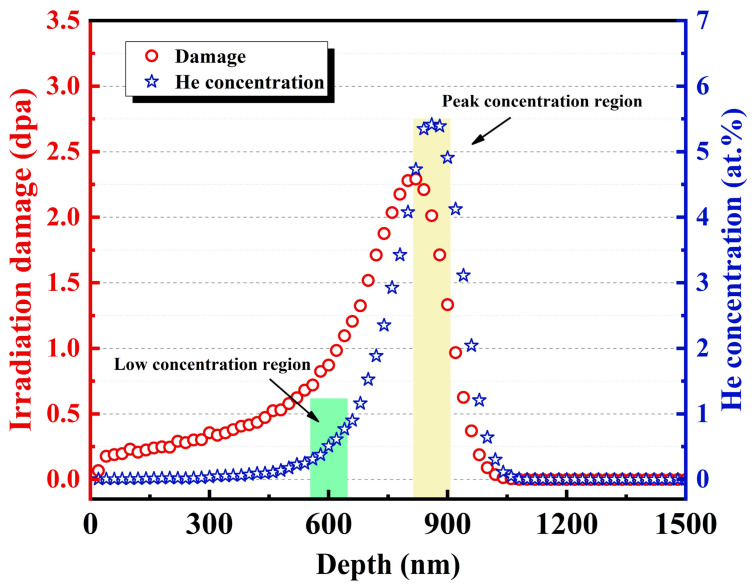
SRIM calculation results of the irradiation damage dose and He concentration as a function of depth for standard 316L stainless steel.

The 400 keV He^+^ ion irradiation experiment was performed on the NEC ion implanter at Xiamen University (Xiamen, China) to a fluence of 1 × 10^17^ ions/cm^2^ at 450 °C (>0.3 *T*_m_, *T*_m_ is the melting point of 316L). The corresponding displacements per atom (dpa) and He concentration as a function of depth were predicted using SRIM-2013 program, as shown in Figure 1. The thin foils for TEM analysis were prepared by using the focused ion beam (FIB) lift-out technique on a FEI Helios G4 workstation. Microstructures of the pristine and ion-irradiated samples were observed by TEM (FEI Cs corrected Titan Cubed Themis G2 operated at 300 kV) in bright field (BF) and high-angle annular dark field (HAADF-STEM) imaging mode. Strain analysis of obtained STEM-HAADF images based on geometric phase analysis (GPA) was performed by Gatan digital micrograph software [16]. Simultaneously, electron energy-loss spectroscopy (STEM-EELS, with camera length of 46 mm and energy dispersion of 0.1 eV/channel) was used to measure the He density of the bubbles in both ion-irradiated SLM 316L and CR 316L samples. In detail, the blue shift of He 1*s* → 2*p* absorption peak [17,18] around 22 eV to 25 eV was collected from the center of individual He bubbles with different diameters of 2–8 nm in the irradiated samples. The step size and pixel time during STEM-EELS measurements was set to be 2.8 Å and 80 ms, respectively, and the electron beam did not damage the bubbles during the EELS measurement. Moreover, the energy resolution of the EELS signal (<0.35 eV at 300 kV) was considered to be the experimental uncertainty in this study.

## 3. Results and Discussion

### 3.1. Microstructures of the Pristine Samples

**Figure 2:**
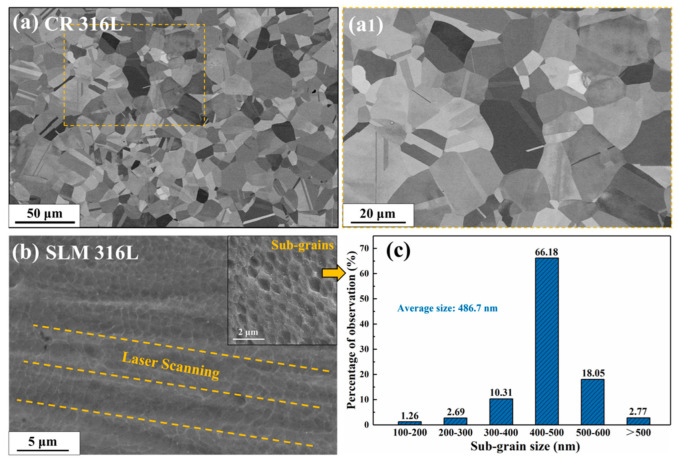
SEM images of (**a**,**a1**) the pristine CR 316L sample, and (**b**) the pristine SLM 316L sample. (**c**) Statistical result of sub-grain size in SLM 316L. Inset in (**b**) shows the typical sub-grains of the SLM 316L sample.

Figure 2a,b are the SEM images of pristine CR 316L and SLM 316L, respectively. Among them, Figure 2(a1) is a high-magnification view of the grains in CR 316L, corresponding to the yellow box area in Figure 2a. The typical equiaxed grains with average size of ~23 μm were observed in CR 316L sample, and the unique cellular sub-grains (SGs) structure can be found in SLM 316L sample, which is consistent with previous reports [8,12]. Based on the SEM images, the distribution and size of SG in SLM 316L were counted and shown in Figure 2c, indicating the sizes of SGs were mainly concentrated between the range of 400–500 nm (66.18% of the total) with an average size of 486.7 nm. In addition, the detectable parallel strips in SLM 316L, which are marked by orange dotted lines in Figure 2b, are considered to be the melt pool boundaries determined by the laser scanning strategy in the manufacturing process.

**Figure 3:**
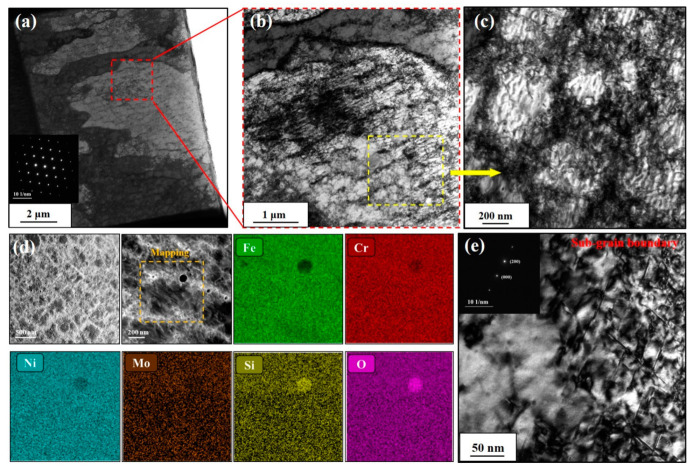
(**a**–**c**) BF-TEM images of the pristine SLM 316L sample. (**d**) The STEM-HAADF images and corresponding EDS mapping results of the sub-grain boundary and oxide particle. (**e**) BF-TEM image with two-beam condition of SGB. Images in (**a**–**c**) were taken near the [011] zone axis, and the image in (**e**) was taken at [011] zone axis with g = 200. The insets in (**a**,**e**) are the SAED pattern of the sample.

Figure 3a–c shows the microstructure with numerous cellular sub-grain boundaries (SGBs) of pristine SLM 316L. Among them, Figure 3b,c are the enlarged view of the red and yellow boxes in Figure 3a,b, respectively, showing the detailed morphology of SGBs in SLM 316L. The average size and distribution of the cellular SGBs observed under TEM are consistent with those in SEM images, suggesting the SGBs in SLM 316L were not damaged or affected by the FIB process. The SAED pattern in Figure 3a shows that the matrix of SLM 316L sample is FCC single-phase structure, which is consistent with the grazing incidence X-ray diffraction (GIXRD) results of the pristine samples (as shown in Figure S2 in the Supplementary Material). Figure 3d shows the STEM-HAADF images and corresponding EDS mapping results of SGBs in the pristine SLM 316L sample.

The results indicate that SLM 316L contains a large number of nano-sized oxide particles with an average diameter less than 50 nm, and the matrix elements exhibit the uniform distribution at the SGBs. Figure 3e is the detailed TEM images of SGBs taken at the two-beam condition of Z = [011] with g = 200, which shows that the SGBs are composed of staggered dislocation network, and the dislocation lines are arranged at angles of 65–70°. The characterization of SGBs and oxide particles via STEM-HAADF and STEM-BF methods can be found in Figure S4 in the Supplementary Material. The formation and evolution of such dislocation network in pristine SLM 316L have been thoroughly studied under the conditions of temperature changes or deformation in previous reports [7,19].

### 3.2. He Bubbles in CR 316L and SLM 316L Samples

**Figure 4:**
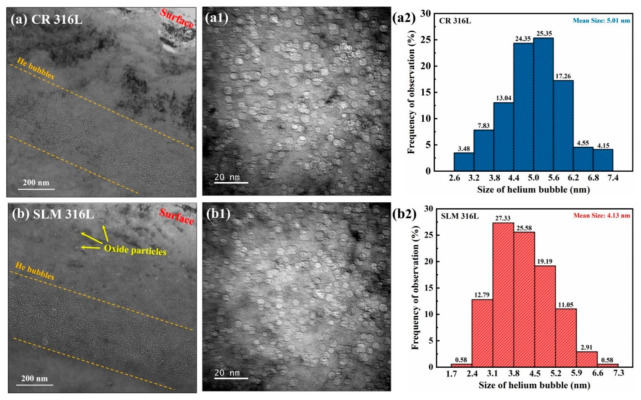
(**a**,**b**) Cross-sectional BF-TEM images of He-irradiated CR 316L and SLM 316L samples. (**a1**,**b1**) Under-focused images with high magnification corresponding to the peak regions of the He concentration in the samples. (**a2**,**b2**) Statistical results for the size distributions of He bubbles in He-irradiated CR 316L and SLM 316L samples, respectively. All the TEM images were taken in the under-focus ~2 μm condition.

Figure 4a,b shows the cross-sectional TEM images of the He-irradiated CR 316L and SLM 316L, respectively. In order to minimize the interference of extra contrast of the dislocations on the observation of He bubbles, the TEM images were acquired at the condition away from the [011] zone axis to a high-order zone axis. The TEM images were taken at under-focus ~2 μm, thus, the He bubbles appear as bright regions.

As shown in the TEM images, the He bubbles are concentrated at the depth of 650–1050 nm from the surface in both types of 316L samples, as marked by the orange dotted line in Figure 3a,b, which are in good agreement with the He concentration peak predicted by SRIM calculations. Figure 4(a1,b1) are the high-magnification images acquired from the peak He concentration region (depth of ~900 nm) of the CR 316L sample and SLM 316L sample, respectively, which show that high-density He bubbles overlap in the matrix. It can be seen from the images that the He bubble size of SLM 316L is smaller than that of CR 316L, suggesting that some features in SLM 316L are different from CR 316L and can inhibit the bubble growth under the same irradiation condition.

To quantify and compare the He bubbles of the two types of 316L samples under the He-irradiation condition, the diameters of the He bubble at the depth range of 750–1000 nm in the two samples were carefully measured and counted, and the statistical results are shown in Figure 4(a2,b2). To avoid mistakes, the statistical data for each sample were derived from at least 10 TEM images, covering an area of about ~0.05 μm^2^. The results show that the diameter of the He bubbles in both samples were close to the Gaussian distribution, and the average diameter of the He bubbles in the observation region was 5.01 nm for the CR 316L sample and 4.13 nm for the SLM 316L sample. Among them, the diameters of the majority of bubbles in the SLM 316L sample were between 3 and 5 nm (accounting for ~72%), while the diameters of bubbles in the CR 316L sample were mainly concentrated between the range of 4–6 nm (accounting for ~70%). Meanwhile, the number densities of the bubbles in the CR 316L sample and SLM 316L sample were estimated to be (6.84 ± 0.22) × 10^23^ m^−3^ and (6.96 ± 0.23) × 10^23^ m^−3^, respectively. The measurement error of bubble density is due to the illegibility of overlapping bubbles and the uncertainty of the sample thickness. Based on the statistical results, under the same He irradiation condition in our study, it was confirmed that the size of the bubbles in SLM 316L are smaller than those in CR 316L, while the difference in bubble densities between the two samples is negligible. The results indicate that the growth and accumulation of the He bubbles in the SLM 316L sample is suppressed, thus showing a smaller average size of bubbles compared to that of the CR 316L sample. These findings are in good agreement with the trends in previous studies [8,13]. Considering that the two types of 316L samples with similar chemical compositions and crystal structures were subjected to the same He irradiation condition, it can be inferred that the formation and growth of bubbles are inhibited due to the differences introduced by the SLM fabrication process.

Understanding the difference in He irradiation resistance between conventional CR 316L and SLM 316L is critical to the future applications of SLM 316L for nuclear systems, and it is important to reveal the specific mechanisms for the formation and accumulation of the He bubbles in SLM 316L from the micro-scale perspective. Based on the characterization of the pristine samples, the possible factors affecting the formation and evolution of He bubbles in SLM 316L are discussed in the next several chapters.

### 3.3. Effects of SGBs on the Bubbles in SLM 316L

The SGBs of SLM 316L were confirmed to be composed of dislocations, which are induced by the thermal expansion/shrinkage in the constrained medium of the fabrication process [4,20]. Simultaneously, the interactions between the irradiation-induced defects and the dislocations [21,22] of SGBs are considered to have considerable influences on the evolution of the He bubbles. Therefore, it is necessary to clarify the contribution of SGBs to the decreased average diameter of the bubbles in the SLM 316L sample compared to that in the CR 316L sample.

**Figure 5:**
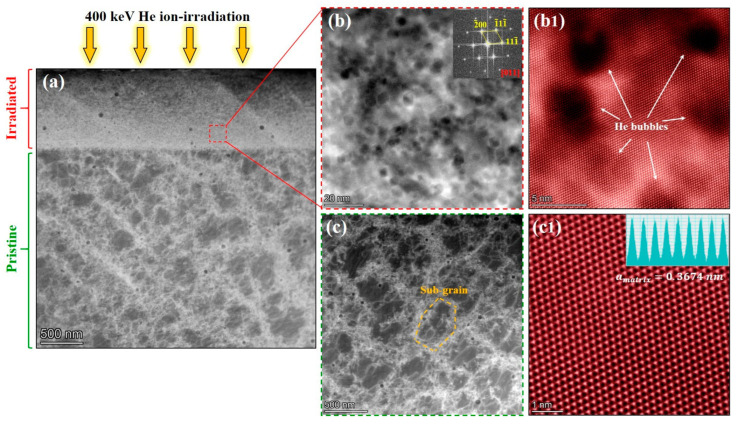
(**a**) Cross-sectional STEM-HAADF image of the He-irradiated SLM 316L sample shows the distinct irradiated layer and pristine region. (**b**,**b1**) The high-resolution images of He bubbles taken from the irradiated layer. (**c**) Survived sub-grains in the He-irradiated SLM 316L sample. (**c1**) The atomic-resolution HAADF image of the matrix in the pristine region, indicating that the lattice constant of the matrix is ~0.3674 nm. The inset in (**b**) is the corresponding FFT pattern.

Figure 5a shows the overall cross-sectional microstructure of the He-irradiated SLM 316L sample, where the irradiated layer (surface layer) and pristine region can be easily identified. Unlike the pristine region, the SGBs in the irradiated layer dissipated and disappeared after He-irradiation. Specifically, as shown in Figure 5b,c, the He bubbles without any phase transformation were detected in the irradiated layer, but SGBs were only found in the pristine region. Considering that the sample as a whole experienced the elevated temperature of 450 °C, the ion-irradiation therefore becomes the only variation between the surface layer and pristine region, suggesting that the disappearance of SGBs is attributed to the He irradiation. Furthermore, as shown in Figure S5 in the Supplementary Material, even though the SGBs diffused or disappeared, the He irradiation did not change the elemental distribution of the irradiated layer. These results indicate that the SGBs in SLM 316L are not stable enough and disappear after an irradiation dose less than 2.5 dpa. Accompanied by the disappearance of the interfaces between SGBs and the matrix, the absorption efficiency of SGBs for irradiation defects should be reduced. Similarly, the diffusion of cellular walls in additively manufactured (AM) 316L under Kr ion irradiation has been recently observed, and the absorption effect of SGBs on irradiation defects has been studied and revealed in detail [9,22].

Moreover, the SGBs undergo dislocation climb after absorbing defects, therefore, causing their diffusion or disappearance, which can explain the phenomenon observed in this study. Based on the findings in the previous research, the SGBs can serve as effective defect sinks, and thus contribute to the inhibition of bubble growth. The growth of He bubbles depends on the accumulation of vacancies and He atoms. The SGBs can trap vacancies and interstitial induced by irradiation and promote their annihilation, thereby reducing the number of available vacancies for bubble absorption. In addition, Figure 5(b1,c1) provides the atomic-resolution HAADF images of the bubbles and matrix, where the lattice constant was measured to be 0.3665 nm and 0.3674 nm, respectively. These results indicate the fact that the irradiation caused a slight decrease in the lattice constant of SLM 316L, which may be due to the stress relief by diffusion or disappearance of SGBs. To summarize this chapter, SGBs can suppress the growth of bubbles by trapping vacancies or small clusters of He-vacancy complexes, but the SGBs are not stable under irradiation, and their effects on bubble growth after the diffusion/disappearance remains an open question and needs to be further investigated by in-situ studies or cluster dynamic calculations [23].

### 3.4. Effects of Nano-Scale Oxide Particles on the Bubbles in SLM 316L

The interfaces can act as efficient sinks for irradiation-induced defects and promote the annihilation or recombination of the defects. This insight has been systematically demonstrated in nano-particle dispersion-strengthened alloys [24,25], multilayer films [26], multiphase alloys [27], and nanocrystalline alloys [28,29]. By analogy, the unique interfaces between oxide particles and the matrix in SLM 316L would be expected to absorb irradiation-induced defects, thereby suppressing the formation and accumulation of the He bubbles. Therefore, investigating the distribution of He bubbles around oxide particles is an effective approach to evaluate the influence of oxide particles on bubble evolution in the SLM 316L sample.

**Figure 6:**
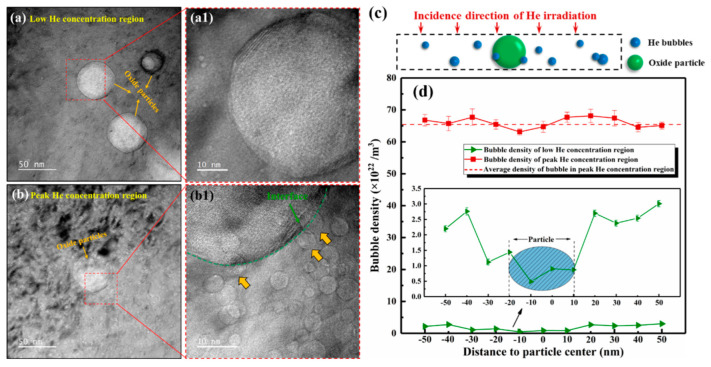
BF-TEM images of He bubbles around the oxide particles in the He-irradiated SLM 316L sample. (**a**,**a1**) Show the low He concentration region (~600 nm depth from surface), (**b**,**b1**) show the peak He concentration region (~850 nm depth from surface), and the orange arrows in (**b1**) indicate bubbles at the interface between oxide particles and the matrix. The above images were taken in the under-focus ~2 μm condition. (**c**) Schematic diagram of bubbles around the particles. (**d**) Densities of the bubbles profiles change near the oxide particles in low and peak He concentration regions.

Figure 6a,b shows the morphology of the oxide particles and the distributions of the surrounding bubbles in the low and peak He concentration regions in He-irradiated SLM 316L, respectively. Notably, the low and peak He concentration regions represent the areas with depths of 550–650 nm and 800–900 nm, corresponding to the SRIM calculation result shown in Figure 1. Results indicate that the size of the oxide particles is in the range of 10–50 nm and the density is about 8.25 × 10^19^/m^3^. In addition, it can be found that the average size and density of the oxide particles in the He-irradiated regions were consistent with those in the pristine regions, suggesting that the He-irradiation process in this study had no observable effect on these particles in SLM 316L. In the dashed box region shown in Figure 6c, the density of bubbles on both sides is counted with a single particle as the center. To avoid mistakes, at least three particles with similar sizes (~30 nm) were selected near the depth of 600 nm and 850 nm for statistical analysis. Figure 6d shows the statistical results of bubble densities around the particles in low and peak He concentration regions.

Two different phenomena were observed in the low He concentration region and the peak concentration region, and their possible mechanisms were explained. Firstly, in the case of the low He concentration, the bubble density is significantly reduced near the particle–matrix interface and within the particle (the green curve in Figure 6d). This trend can be explained by the promoted defect annihilation at the interfaces between the particles and matrix. Specifically, the irradiation-induced point defects tend to migrate to the interface and annihilate here, resulting in a decrease in the defect concentration around the particles, and thus creating the denuded zone [30] of He bubbles. Secondly, however, the effects of the interfaces on bubbles are significantly diminished in the case of the peak He concentration region, which is manifested by the fact that the density of the bubbles around the particles is consistent with that in the matrix (the red curve in Figure 6d). This finding may be attributed to the excessive defect concentration and the low local density of oxide particles in the peak damage region. On one hand, it is undeniable that part of irradiation defects annihilate and recombine at the interface between the oxide particles and matrix, but the recovery of such defects may be negligible because the defect concentration at the peak damage region far exceeds the absorption capacity of the interfaces. On the other hand, the low local density of the particles is not sufficient to have a significant absorption effect on irradiation defects. In this case, the migration and accumulation of vacancies and He atoms play a more significant role in bubble formation and growth. Under the irradiation condition of this study, the oxide particles with low local density may, therefore, not be efficient sinks for irradiation defects, nor the main factor that dominates the decreased bubble diameter in SLM 316L compared to that in CR 316L.

### 3.5. Difference in He Density inside the Bubbles between CR 316L and SLM 316L

The He density (or He pressure) inside the bubbles could modify the accumulation process of He atoms, and therefore influence the formation and growth of He bubbles [18,31]. On this basis, interestingly, the above characterization results show that the He bubbles in the SLM 316L sample are all nearly spherical, while some square-like or ellipsoidal He bubbles exist in the CR 316L sample, as shown in Figure S6 in the Supplementary Material. Notably, bubbles with different shapes were observed using TEM in the same axial direction of near *Z* = [011]. This result suggests that the He densities (or He pressure) inside the bubbles in the CR 316L and SLM 316L may be different, resulting in the differences in the shape of the He bubbles. Therefore, it is necessary to understand the effects of the He density inside the bubbles on their formation and growth in the two types of 316L samples. The feasible approach to quantify the He density of nano-sized He bubbles is to collect and evaluate the 1*s* → 2*p* transition of ^3^He via EELS [17,18,32]. Specifically, the absorption peak of a free He atom is located at 21.22 eV, while the absorption peak of a He atom within the bubble exhibits a detectable increase in energy loss. The blue shift in the absorption peak is due to the overlapped effect of the wave-functions of adjacent He atoms [17]. Therefore, the function of He density and shift in the absorption peak can be described as Equation (1) [18,33].

(1) $$\Delta E_{peak}=C\cdot n_{He}$$

where $\Delta E_{peak}$ represents the energy blue shift of the He absorption peak (eV), $n_{He}$ is the He density at the test site (He/nm^3^), and C is the constant describing the correspondence between energy shift and He density. Based on a previous report [17], the value of C is between 0.015 to 0.044 (eV·nm^3^), and C=0.035 eV·nm^3^ was used to measure the He density in this study. Notably, different C values significantly affect the calculation results of He density. However, it is reasonable and convincing to quantify and compare the relative trend of He densities between the CR 316L and SLM 316L samples under the same C value condition. Furthermore, considering the significance of the overlap of the electron wave-functions and high densities of He atoms, the He pressure inside bubbles with the hard-sphere model can be evaluated by Equation (2) [17] in this study.

(2) $$P=n_{He}kT\left(\frac{1+y+y^{2}-y^{3}}{{\left(1-y\right)}^{3}}\right),\;y=\frac{\pi n_{He}d_{s}^{3}}{6}$$

where $n_{He}$ is the atomic number density of He, k is the Boltzmann constant, y is the packing ratio, and $d_{s}$ represents the hard-sphere diameter, which is closely related to temperature. Based on the equation, obviously, when the temperature and diameter of the bubbles are the same, the pressure is proportional to the He density inside the bubble.

**Figure 7:**
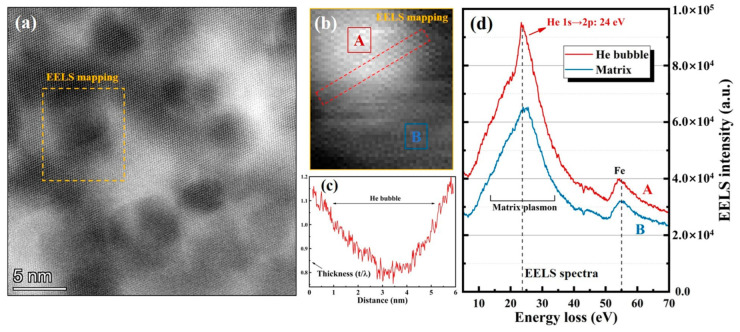
(**a**) Typical STEM-HAADF image of He bubbles in the SLM 316L sample. (**b**) EELS spectrum image (SI) obtained from the region of EELS mapping in (**a**). (**c**) Thickness profile corresponding to the red box of (**b**) measured via EELS SI. (**d**) EELS profile collected from the region A (red curve) and region B (blue curve) in (**b**).

Figure 7a shows the typical STEM-HAADF image of a He bubble in SLM 316L acquired from the peak He concentration region, and the bubbles appear as low intensity in contrast. Figure 7b is the high-loss EELS spectrum image (SI) corresponding to the orange dotted box region in Figure 7a, where the He bubbles appear as bright regions. As shown in Figure 7c, the relative thickness (t/λ) profile corresponding to the red box of Figure 7b was measured by using low-loss EELS SI, indicating that the relative thickness at the bubble is significantly smaller than at the matrix. This is because the bubbles are equivalent to filling the cavities with He atoms, and the existence of the cavities would lead to the decrease in thickness measured by EELS data. Figure 7d shows the corresponding signals collected from the center of the bubble (point A for red curve) and the matrix (point B for blue curve).

The EELS signal collected from the matrix shows two detectable peaks at 25 eV and 55 eV, which are labeled as the absorption peak of the Fe element (55 eV) and the excitation of matrix plasmon peak (25 eV). In the case of the signal collected from the bubble center, the unique He 1*s* → 2*p* transition peak at 24 eV was detected and identified as well as the above explained plasmon peaks. The result validates the feasibility of collecting the He absorption peak via the EELS spectrum, and it provides the example method for acquiring the peak energy value of the He 1*s* → 2*p* transition for a single bubble in this study. Notably, more necessary EELS measurement results of the He bubbles with different diameters can be found in Figures S7 and S8 in the Supplementary Material.

**Figure 8:**
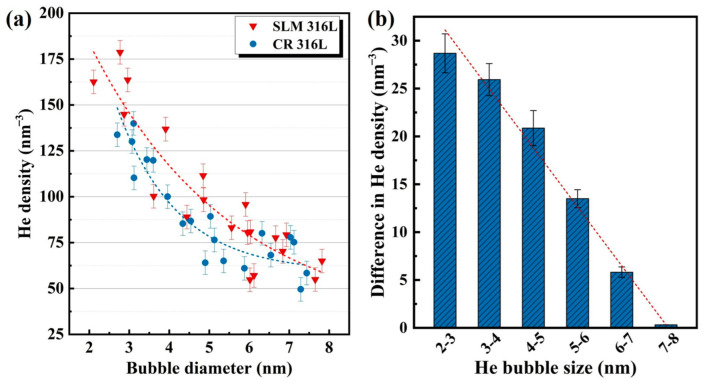
(**a**) Calculated He densities for single He bubbles with different diameters of 2–8 nm in the CR 316L sample and SLM 316L sample. (**b**) The differences in He densities of bubbles with similar size between the CR 316L sample and SLM 316L sample.

To accurately quantify and compare the He densities inside the bubbles with different diameters in the CR 316L and SLM 316L samples, the EELS spectrum of at least 20 bubbles in each sample were carefully collected and counted. The Gaussian function fitting was used in the collected EELS spectra for obtaining the precise value of the He peak energy in both types of 316L sample. Figure 8a shows the statistical results of the determined He density as a function of bubble diameter in the two types of 316L samples. It can be found that the measured He density within each single bubble decreases with increasing the diameter of the bubble, which follows the Laplace–Young’s law reported in previous research [33,34], as shown in Equation (3). That is, the bubble pressure or He density is proportional to the bubble surface intension and stress of the surrounding matrix, while it is inversely proportional to the bubble size. In addition, for the bubbles with similar diameters, it can be found that the He densities inside the bubbles in the SLM 316L sample are generally higher than those in CR 316L sample. For example, the average He density of the bubbles in the 2–4 nm diameter range was measured to be ~147 nm^−3^ for the SLM 316L sample and ~125 nm^−3^ for the CR 316L sample. Based on previous research [35,36], the growth of He bubbles is mainly achieved by the migration/coalescence of small-sized bubbles and the accumulation of vacancies/He atoms in large-sized bubbles. In this study, on one hand, the high He density within bubbles of the SLM 316L sample may reduce the mobility of the bubbles by slowing the vacancy surface diffusion [37], and therefore inhibit the bubble growth via the coalescence of small bubbles. On the other hand, the ability of He bubbles to capture vacancies is negatively related to the He density in the bubbles [38]. Thus, the higher He density within the He bubbles of SLM 316L could also suppress the growth of bubbles by reducing the reabsorption of vacancies.

Furthermore, Figure 8b shows the difference in He density of the bubbles with different diameter ranges in the two samples, which suggests that the He density of bubbles in SLM 316L is visibly higher than that of CR 316L under the condition of small-sized (<6 nm) He bubbles; however, in the case of large-sized (>6 nm) bubbles, the measured He densities in the SLM 316L sample are close to those in the CR 316L sample. This intriguing trend may provide further evidence that the effects of He density on bubble growth dominate in bubbles with a small size (<6 nm), whereas the suppression effects of bubble growth are no longer significant when the bubble size grows to a certain value of ~6 nm (or after the He-irradiation dose reaches a certain level).

**Figure 9:**
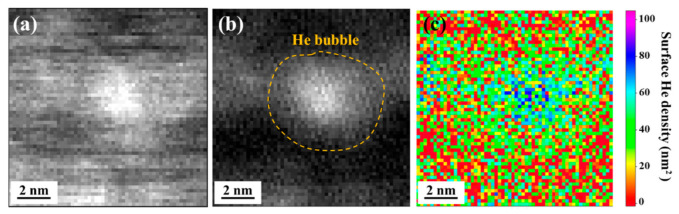
(**a**) Original EELS signal collected around the bubble. (**b**) EELS signal between 22 and 25 eV without matrix. (**c**) Surface He density map calculated in He/nm^2^.

Figure 9 shows the EELS spectrum images and determined surface He density of the bubbles in the SLM 316L sample after data treatment [39,40]. Among them, the convergence and collection angles were set to 30 and 5.9 mrad, respectively, and the cross-section $\sigma_{He}=5$.1 × 10^−6^ nm^2^ was calculated using the Sigmak-3 program [41]. Figure 9a shows the original spectrum image collected around the He bubble of SLM 316L, and it is difficult to clearly distinguish the boundary between the bubble and the matrix from the image. To minimize the interference of the matrix on the bubble, the EELS signal needs to be filtered. The white pixels in Figure 9b represent a high intensity EELS signal between 22 and 25 eV, corresponding to the higher density of ^3^He in the bubble than that in the matrix.

Obviously, the distribution of He density within a single bubble exhibits the trend of being highest in the center and gradually decreasing as far from the center, which is due to the fact that the beam encounters less He atoms at the edge of the bubble [39]. Moreover, the determined surface He densities show that the maximum He density of the bubble with a diameter of ~4.5 nm is 90 nm^−2^, which is in good agreement with the above He density calculated using Equation (1); however, the average He density within this bubble was estimated to be ~70 nm^−2^. These results indicate that the 2D information (surface He density) and the calculations for 3D sample are well correlated for the maximum He density within a single bubble. However, it is worth noting that the average He density of the bubble may significantly lower than the maximum value.

(3) $$P_{bubble}=\frac{2\sigma }{r}+s$$

Equation (3) shows the general form of Laplace–Young’s law, where σ is surface tension of the bubble, r represents the bubble radius, and s is the bubble surface stress [33]. Combining the measured He densities with the Laplace–Young’s law, it can be deduced that the tension (σ) of bubble surface and stress (s) of surrounding matrix are contributory to the increased He density (or pressure) within the bubbles in SLM 316L compared to the CR 316L sample. Under the same chemical composition and irradiation conditions (temperature and irradiation dose), the bubble surface tension (σ) in CR 316L and SLM 316L can be considered to be consistent, whereby the stress (s) of the surrounding matrix is the main factor that affects the He densities of bubbles in the two 316L samples.

**Figure 10:**
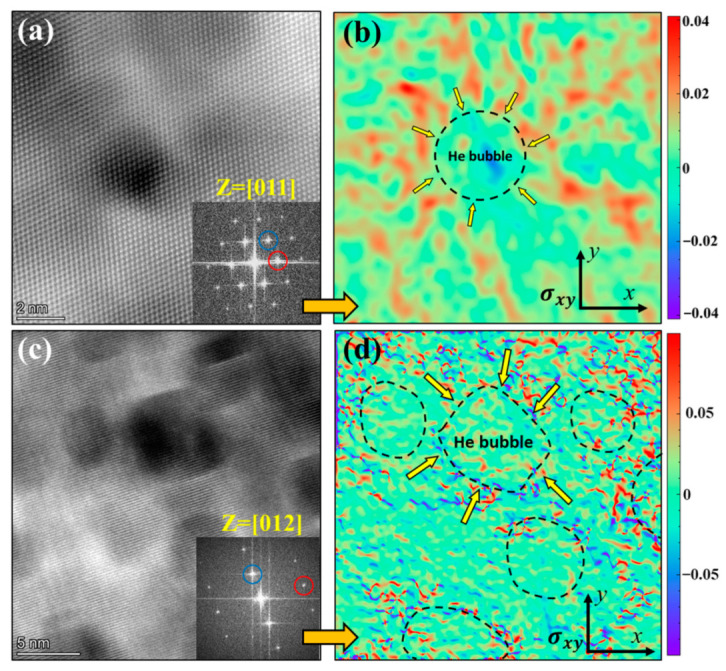
STEM-HAADF images of He bubbles in the SLM 316L sample taken at (**a**) Z = [011] zone axis and (**c**) Z = [012] zone axis. (**b**,**d**) GPA strain mapping of the *σ_xy_* strain field around the He bubbles corresponding to (**a**,**c**), respectively. The red and blue circles refer to the selected minimum vector g during the GPA calculations.

Figure 10b,d provide the calculated strain mappings corresponding to the atomic resolution HAADF images of the bubbles in SLM 316L shown in Figure 10a,c. Among them, Figure 10a,c were taken at the Z = [011] and Z = [012] zone axis, respectively, to evaluate the matrix stress from two different directions (see the FFT patterns in Figure 10a,c). The results demonstrate the obvious stress fields around the He bubbles, which are directed from the matrix to the center of the bubbles. In addition, the magnitude of the detected stress field around the bubble becomes weaker with the increase in the diameter of the bubble, which is in good agreement with the trend of determined He densities inside the bubbles. However, such significant stress fields around the bubbles were not detected in the CR316L, confirming that the increased He densities of bubbles in SLM 316L are due to the unique matrix stress fields introduced by the manufacturing process.

**Figure 11:**
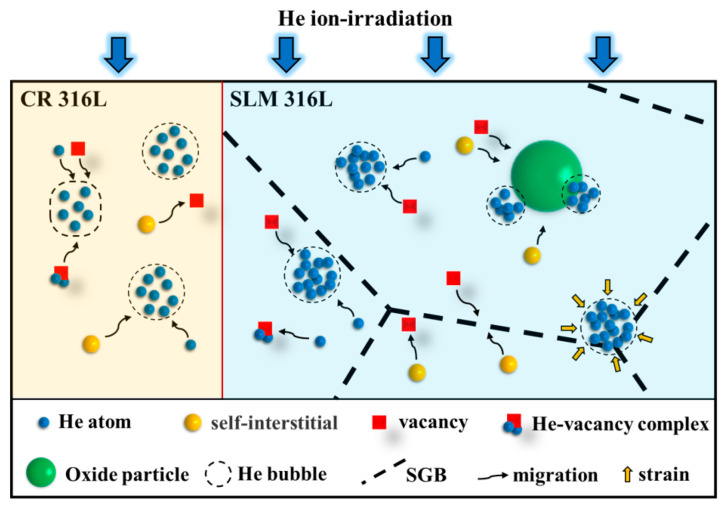
Schematic illustration of bubble formation and growth in He-irradiated CR 316L and SLM 316L samples.

Finally, as shown in Figure 11, the mechanism for the inhibition of bubble growth in the SLM 316L sample can be analyzed accompanied by the effects of SGBs, oxide particles, and He densities within the bubbles. In the early stages of irradiation (low irradiation dose), the SGBs are considered to be the most critical factor for inhibiting bubble growth due to the significant defect trapping effects. However, since the SGBs are not stable under irradiation, the main factor inhibiting bubble growth may be replaced by the increased He densities within the bubbles in the later stage of irradiation. In addition, the absorption effect for He atoms of the interfaces between the oxide particles and matrix is effective at low He concentration regions, while that effect on high-density bubbles is negligible in this study owing to the relatively low irradiation temperature (450 °C) and low local density of the particles.

## 4. Conclusions

In summary, a series of experimental studies were conducted on He bubbles in SLM 316L and CR 316L. It was found that SLM 316L exhibits improved He-irradiation resistance in suppression of bubble growth compared with conventional CR 316L, and the possible factors and internal mechanisms were systematically discussed. The main conclusions of this study are presented as follows:

1. Under the same He irradiation condition, the average diameter of He bubbles in the SLM 316L sample is smaller than that in the CR 316L sample, while the bubble densities are basically consistent in both types of 316L.
2. The effects of unique sub-grain boundaries have primary contributions to the decreased bubble diameter in SLM 316L, especially at low irradiation dose conditions. However, the nano-sized oxide particles with low local density may not be efficient sinks for irradiation defects, nor the main factor that dominates the decreased bubble diameter in SLM 316L for the irradiation condition in this study.
3. The detectable differences in He densities inside the bubbles were found in SLM 316L and CR 316L via EELS measurements. The inhibition effects of increased He density on bubble growth were explained as two mechanisms: Firstly, increased He densities have an inhibition effect on the coalescence of small bubbles. Secondly, high He densities can stabilize the bubbles and reduce reabsorbed vacancies.
4. The differences in He density inside the bubbles with different diameters in SLM 316L and CR 316L follow a linear deceasing trend, which is attributed to the combination of the Young–Laplace law and internal stress fields in SLM 316L. It indicates that, in SLM 316L, the stress-dominated increase in He density is more significant in small-sized bubbles than that in bubbles with large sizes.

## Figures and Tables

**Table 1:** Chemical compositions (wt. %) of the CR 316L and SLM 316L.

	Fe	Cr	Ni	Mo	Mn	Si	C	S/P
CR 316L	Bal.	16.94	10.50	2.07	1.18	0.29	0.02	<0.01
SLM 316L	Bal.	17.22	10.15	2.25	1.06	0.43	0.02	<0.01

## Data Availability

Data are available upon request.

## Supplementary Materials

The following supporting information can be downloaded at: https://www.mdpi.com/article/10.3390/ma16113922/s1.
