# Supplementary material for: The Mechanisms of Inhibition Effects on Bubble Growth in He-Irradiated 316L Stainless Steel Fabricated by Selective Laser Melting
Source: Materials (Basel). 2023 May 24;16(11):3922. doi: 10.3390/ma16113922 (PMC10253493; doi:10.3390/ma16113922)
Supplement: Supplementary file 1 [file materials-16-03922-s001.zip › materials-2383841-supplementary.pdf]

Supplimentary Material for

The mechanisms of inhibition effects on bubble growth in He-irradiated 316L stainless steel fabricated by selective laser melting

Shangkun Shen[1], Zhangjie Sun[2], Liyu Hao[1], Xing Liu[1], Jian Zhang[3], Kunjie Yang[4], Peng Liu[5], Xiaobin Tang[2], Engang Fu[1,*]

[1] *State Key Laboratory of Nuclear Physics and Technology, Department of Technical Physics, School of Physics, Peking University, Beijing, 100871, China;*

[2] *Department of Nuclear Science and Technology, Nanjing University of Aeronautics and Astronautics, Nanjing, 211106, China;*

[3] *College of Energy, Xiamen University, Xiamen, 361005, China;*

[4] *College of Nuclear Equipment and Nuclear Engineering, Yantai University, Yantai 264005, China;*

[5] *Institute of Frontier and Interdisciplinary Science and Key Laboratory of Particle Physics and Particle Irradiation (MOE), Shandong University, Qingdao 266237, China;*

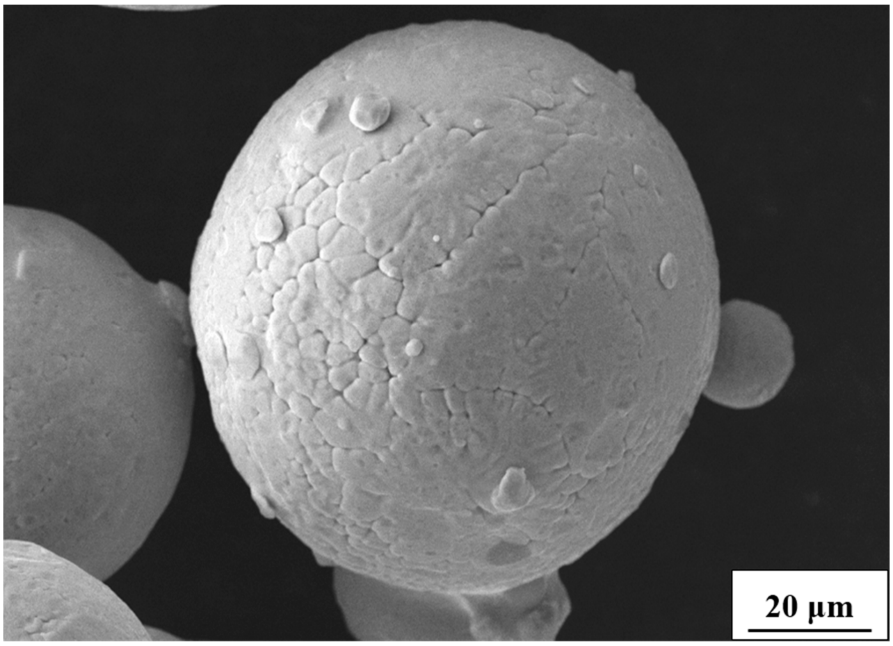

Fig. S1. SEM image of the AISI 316L powder after 24 h of ball milling

The essence of selective laser melting (SLM) technology is laser melting of powder materials, hence, the powder with different particle sizes or morphologies can have a significant impact on the quality of the final formed bulk material.

The AISI 316L raw powder used in this study was directly purchased from the ZhongNuo

Advanced Material（Beijing）Technology Co., Ltd, which may have several issues such as uneven size and non-spherical particle shape that are not conducive to SLM preparation. Based on our previous research experience, appropriate ball milling treatment for the purchased raw powder materials is expected to achieve better preparation results. Especially, ball milling in an argon atmosphere for 24 hours can achieve the best particle sphericity and size uniformity. As shown in Fig. S3, uniform spherical AISI 316L powders with average size of ~50 μm were carefully prepared via ball milling and used for the SLM processes in this study

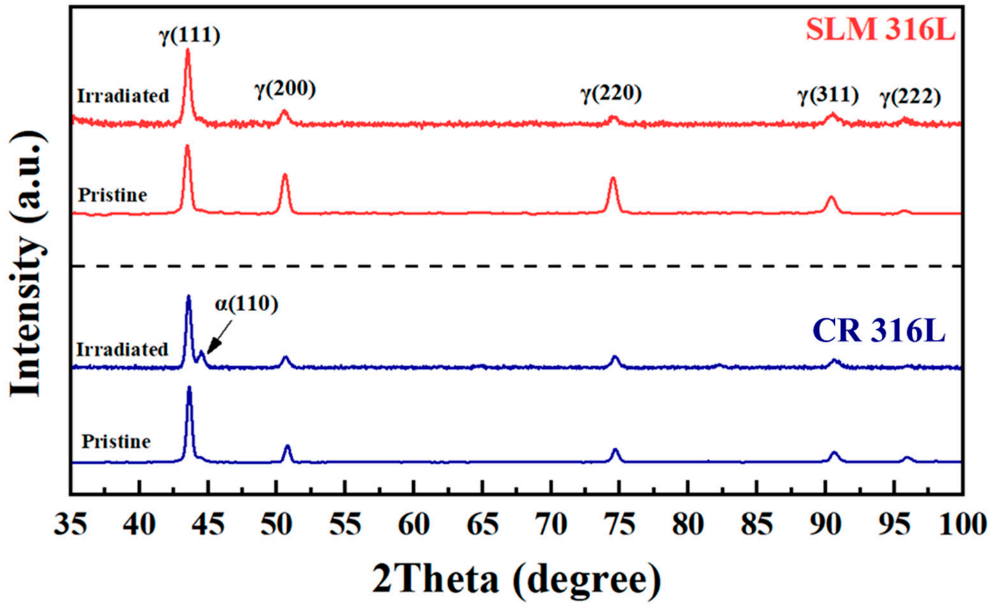

Fig. S2. Grazing incidence x-ray diffraction (GIXRD) patterns of the two types of 316L samples before and after He-irradiation.

The penetration depth of GIXRD was adjusted to approximately 1.5 μm by fixing the incident beam at 1.5° relative to the sample surface. Several FCC austenite diffraction peaks of γ-(111), γ-(200), γ-(220), γ-(311), and γ-(222) are detectable in the pristine samples. However, the CR 316L sample shows a α-(110) diffraction peak after the irradiation, suggesting that the part of original FCC phase of the CR 316L sample has changed to ferrite or martensite after irradiation, while the SLM 316L remained fully crystalline and original FCC phase. This result demonstrated that the austenite phase of SLM 316L has higher stability under He-irradiation conditions compared to that of CR 316L.This finding is consistent with previous reports.

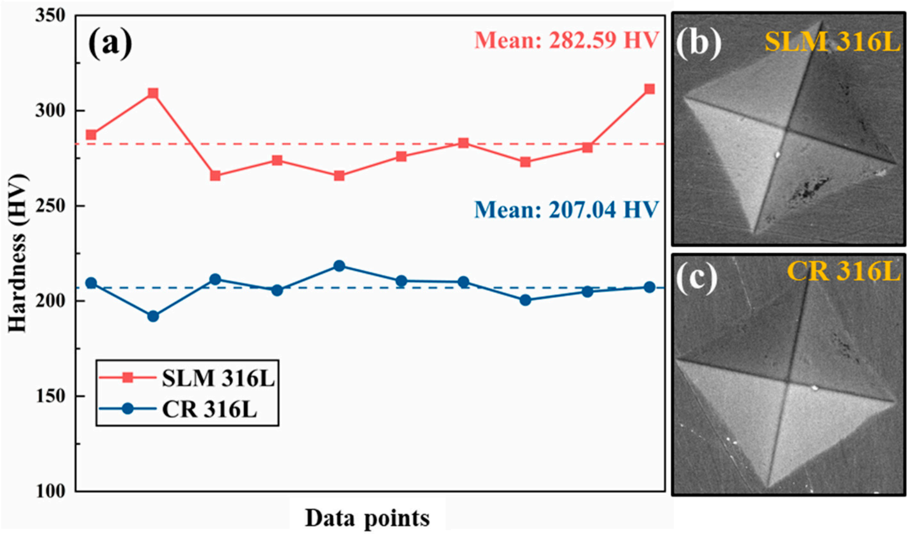

Fig. S3. (a) Vickers hardness test results for pristine SLM 316L sample and CR 316L sample. (b) and (c) show the Vickers indentation of SLM 316L and CR 316L, respectively.

Vickers hardness measurements were carried out on the pristine SLM 316L sample and CR 316L sample after electrochemical polishing, and each sample was tested 10 times on flat and scratch-free areas. The main hardness of SLM 316L and CR 316L is measured to be 282.59 HV and 207.04 HV. The hardness of SLM 316L is increased by about 36.5% compared to CR 316L. The shapes of Vickers indentations of the two types of 316L samples are consistent, and no detectable crack initiation is observed.

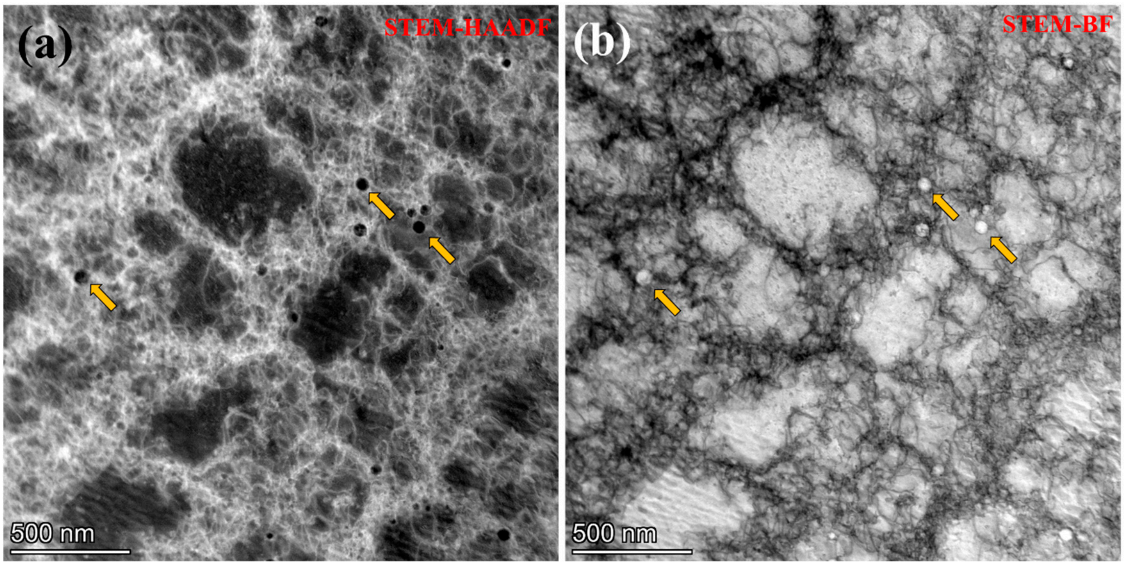

Fig. S4. Micromorphology of the sub-grains in pristine SLM 316L sample. (a) STEM-HAADF image. (b) STEM-BF image. The yellow arrows indicate the unique nano-sized oxide particles.

SLM as one of the additive manufacturing (AM) methods, whose main principle is the quick melting of powders and the rapid solidification of liquids in a layer-by-layer manner to efficiently fabricate a given component. During this process, the deformation induced by thermal expansion/shrinkage in the sample can be considered as the primary source of dislocations in AM materials. In addition, the micro-segregation in AM materials has been found at the subgrain boundaries (SGbs), which can also be considered as a promoting factor for the formation of unique SGbs. In summary, the thermal stress contained in SLM materials is not only the source of their irradiation resistance, but also the main factor that generates SGBs. However, after He irradiation, due to the introduction of a large number of bubbles/irradiation defects, the thermal stress was released. Correspondingly, we observed the dissipation of the SGBs in irradiated regions after He-irradiation in our work.

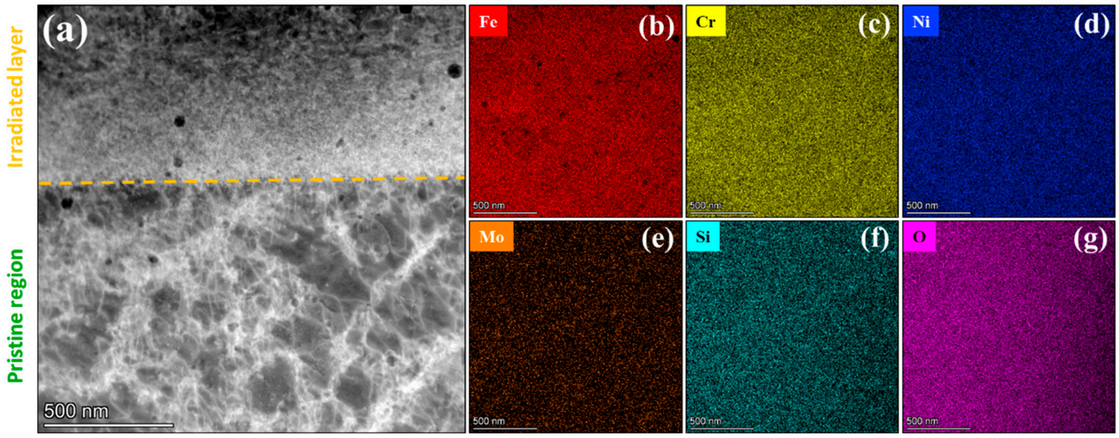

Fig. S5. (a) HAADF image of He-irradiated SLM 316L sample, where the irradiated layer and pristine region can be easily identified. (b)-(g) corresponding EDS mapping results of the sample, which indicate the elements distributions of irradiated layer and pristine region are consistent.

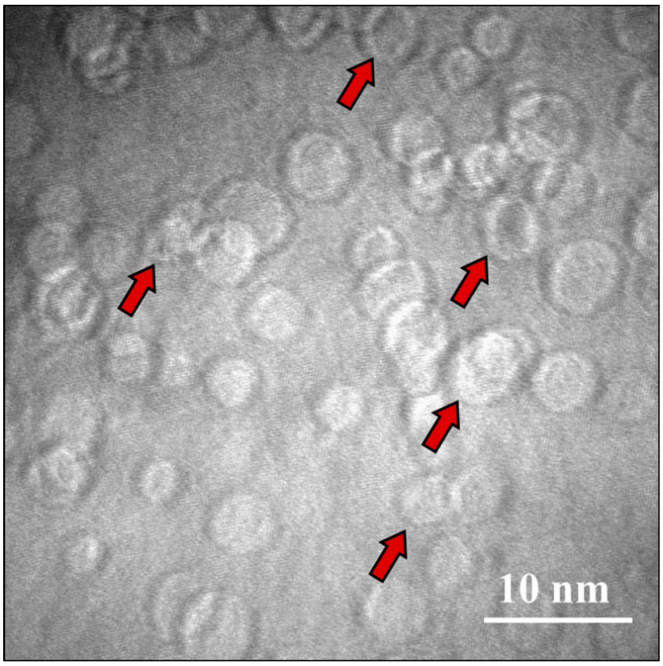

Fig. S6. TEM image of the He bubbles in CR 316L. The bubbles indicated by the red arrow appear as ellipsoids

The shape of He bubbles depends on both the axial direction captured by TEM and the pressure inside the bubbles (or the ratio of He atoms to vacancies). In fact, we detected some ellipsoidal bubbles (or approximate square shape) in CR 316L near Z=[111] axis, as shown in Fig. S5, but this phenomenon was not observed in SLM 316L. Therefore, the difference in shapes of the bubbles in CR 316L and SLM 316L can be used as a confirmation of He density results in this study.

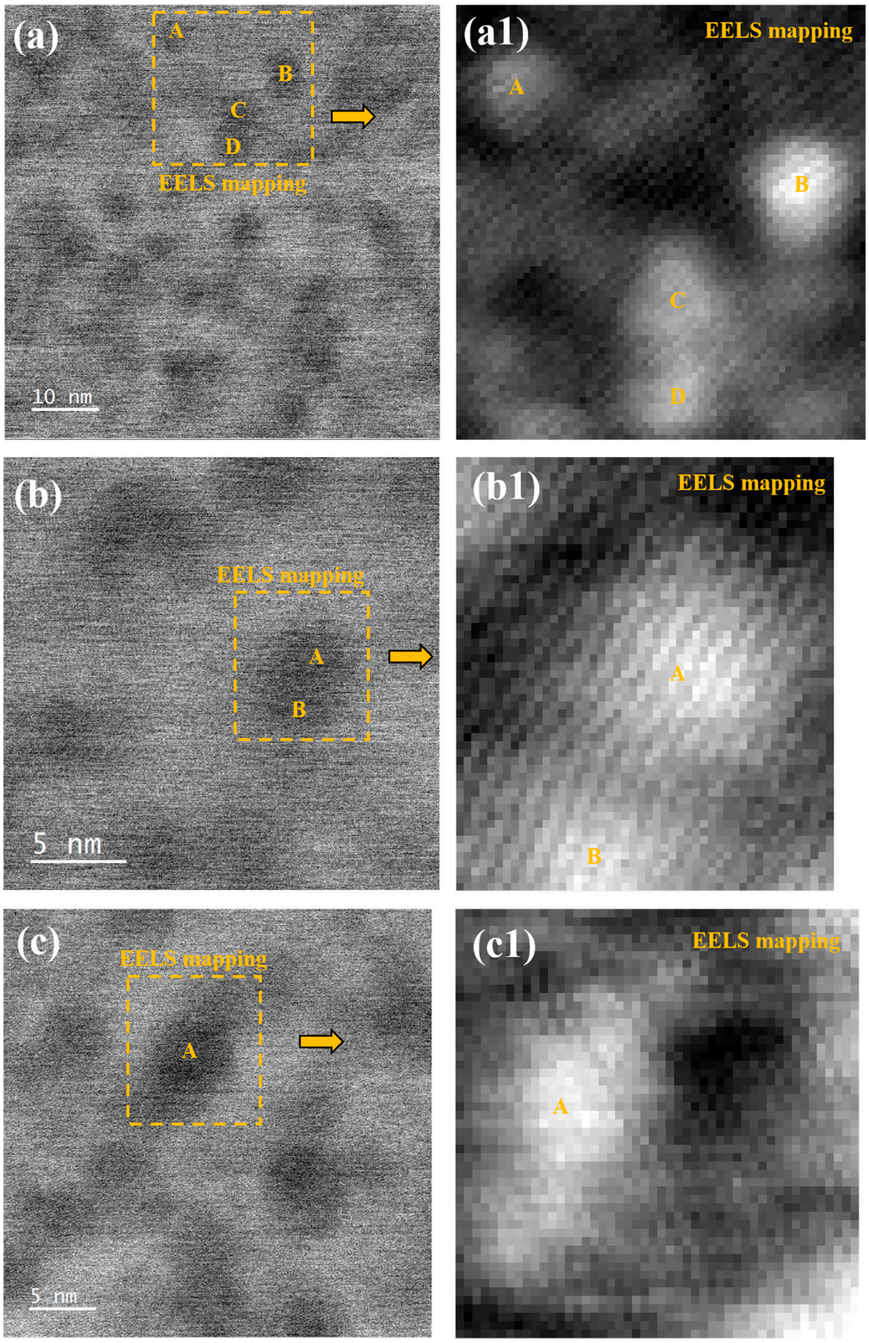

Fig. S7. HAADF images and corresponding EELS spectrum images. (a)-(a1) SLM 316L sample, (b)-(c1) CR 316L sample. Notably, the EELS spectrum images were derived from the signals between 22-25 eV.

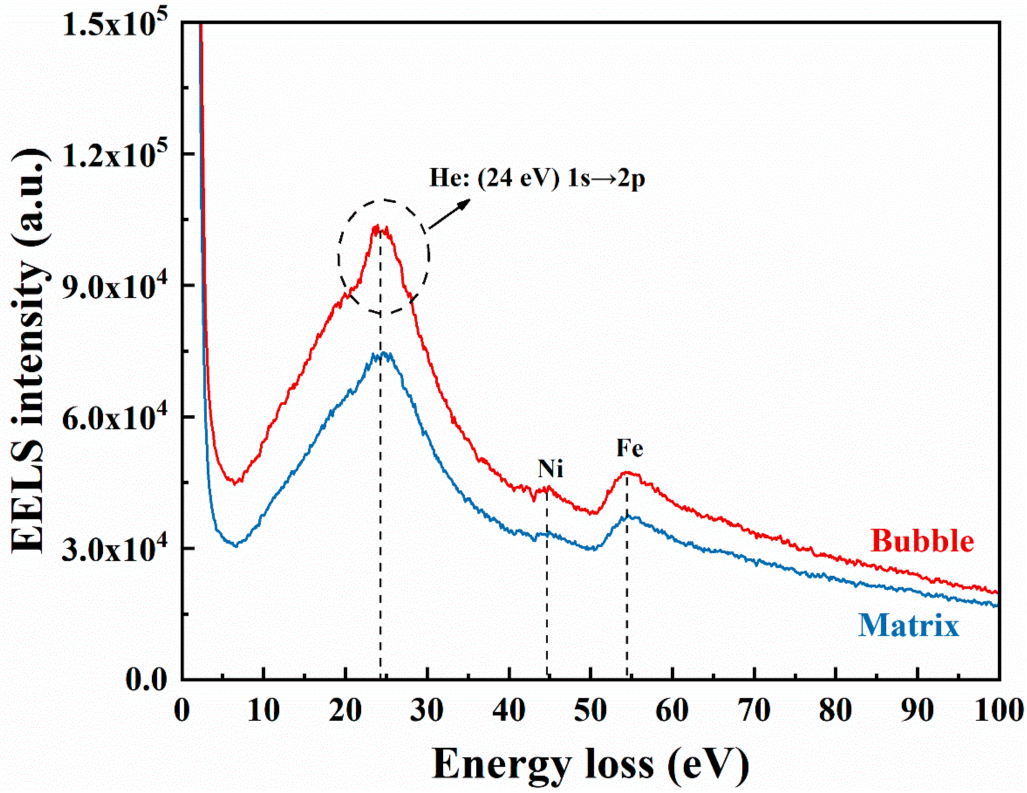

Fig. S8. EELS profile collected from the matrix (blue curve) and He bubble (red curve) in He-irradiated CR 316L sample.

The absorption peak (at ~24 eV) circled by the dotted circle in the figure shows the He 1s→2p transition. In addition, the absorption peaks of Ni and Fe were easily identified at ~44 eV and ~54 eV, respectively.
